# Modulation of High-Intensity Optical Properties in CdS/CdSe/CdS Spherical Quantum Wells by CdSe Layer Thickness

**DOI:** 10.3390/nano14191568

**Published:** 2024-09-27

**Authors:** Wenbin Xiang, Chunzheng Bai, Zhen Zhang, Bing Gu, Xiaoyong Wang, Jiayu Zhang

**Affiliations:** 1 Advanced Photonics Center, School of Electronic Science and Engineering, Southeast University, Nanjing 210096, China; 230208149@seu.edu.cn (W.X.); 230238398@seu.edu.cn (C.B.); gubing@seu.edu.cn (B.G.); 2 National Laboratory of Solid State Microstructures, College of Engineering and Applied Sciences, and School of Physics, Nanjing University, Nanjing 210093, China; dz1922040@smail.nju.edu.cn (Z.Z.); wxiaoyong@nju.edu.cn (X.W.)

**Keywords:** spherical quantum wells, Auger recombination, multiexciton properties, amplified spontaneous emission, optical nonlinearity

## Abstract

Spherical quantum wells (SQWs) have proven to be excellent materials for suppressing Auger recombination due to their expanded confinement volume. However, research on the factors and mechanisms of their high-intensity optical properties, such as multiexciton properties and third-order optical nonlinearities, remains incomplete, limiting further optimization of these properties. Here, a series of CdS/CdSe (xML)/CdS SQWs with varying CdSe layer thicknesses were prepared. The modulation effects of CdSe shell variations on the PL properties, defect distribution, biexciton binding energy, and third-order optical nonlinearities of the SQWs were investigated, and their impact on the material’s multiexciton properties was further analyzed. Results showed that the typical CdS/CdSe(3ML)/CdS sample exhibited a large volume-normalized two-photon absorption cross-section (18.17 × 10^2^ GM/nm^3^) and favorable biexciton characteristics. Optical amplification was observed at 12.4 μJ/cm^2^ and 1.02 mJ/cm^2^ under one-photon (400 nm) and two-photon (800 nm) excitation, respectively. Furthermore, different amplified spontaneous emission spectra were observed for the first time under one/two-photon excitation. This phenomenon was attributed to thermal effects overcoming the biexciton binding energy. This study provides valuable insights for further optimizing multiexciton gain characteristics in SQWs and developing optical gain applications.

## 1. Introduction

Low-dimensional II–VI semiconductor nanomaterials exhibit unique physical and chemical properties due to quantum confinement effects [1,2]. Among these, quasi-zero-dimensional (0D) quantum dots (QDs) show great potential in next-generation lighting and display technologies due to their tunable photoluminescence, narrow-band emission, and high PL quantum yield [3,4,5]. However, while strong quantum confinement effects provide these advantages, they also increase the probability of non-radiative Auger recombination of carriers, limiting the development of quantum dots in high-intensity optical applications [6,7].

Research has shown that the Auger lifetime in single-material quantum dots is approximately linearly correlated with their volume, i.e., $\tau_{Auger}\propto V\;$[8]. Subsequent studies have further extended the applicability and precision of this correlation, but the positive relationship between Auger lifetime and material volume remains widely accepted [9,10,11]. Therefore, by modifying the morphology of low-dimensional semiconductor nanomaterials and transforming quasi-zero-dimensional (0D) quantum dots into one-dimensional nanorods [12,13] or two-dimensional nanoplatelets [14,15], it is possible to partially relieve geometric constraints while maintaining quantum confinement effects, thereby achieving extended biexciton emission lifetimes as the confined volume increases. Another effective strategy is to introduce core/shell structures, which, through carefully designed band engineering, reduce electron–hole wavefunction overlap while suppressing carrier trapping by surface defects, thus effectively inhibiting Auger recombination and significantly enhancing the material’s high-intensity optical properties [16,17]. Klimov et al. first demonstrated large-shell CdSe/CdS QDs, which exhibit a substantial reduction in Auger processes [18].

Based on these considerations, researchers have begun to focus on novel nanomaterials that simultaneously satisfy the requirements of expanding the confined volume and introducing core/shell structures, such as colloidal core/shell nanoplatelets and spherical quantum well structures. These materials have demonstrated significant advantages in suppressing Auger recombination and enhancing high-intensity optical properties (such as biexciton characteristics) [19,20]. Kunneman et al. successfully achieved an ultra-long Auger recombination lifetime of about 10 ns by coating 4ML CdSe nanoplatelets with CdS/ZnS shell layers, highlighting the synergistic effect of large confined volume and core/shell structure [19]. Mikhail et al. achieved near-complete suppression of Auger recombination in CdS/CdSe/CdS spherical quantum well structures, exhibiting ultra-long optical gain lifetimes (>6 ns) and excellent biexciton quantum yields (81%) [21]. They further investigated the effect of CdS core size on biexciton quantum yield, achieving an average biexciton quantum yield of up to 82% in samples with large CdS cores of 8.2 nm in diameter [22]. Expanding on this work, the group continued to investigate the influence of CdS core size on various aspects of quantum structure performance. They obtained large two-photon absorption cross-sections of 0.4 × 10^6^–7.9 × 10^6^ GM and multimodal amplified spontaneous emission in samples with different CdS core sizes, with amplified spontaneous emission thresholds as low as 1 mJ/cm^2^, demonstrating excellent optical properties [23]. Building on the suppression effect of enlarged CdSe confinement layer volume on Auger recombination, X-ray excited scintillation studies showed long Auger–Meitner lifetimes (>10 ns) and radioluminescence lifetimes of 2.5 ns, further confirming this suppression effect [24]. To further optimize performance, they introduced a ZnS blocking layer outside the CdS/CdSe/CdS spherical quantum well, effectively inhibiting surface carrier decay, achieving τ_2, Auger_ = 110.2 ns in 8.7 nm core QSs, one of the longest Auger lifetimes reported so far in colloidal NCs [25].

However, it should be noted that the introduction of shell layers not only affects Auger recombination but also alters the material’s energy-level structure and quantum confinement effects, thereby influencing other high-intensity optical properties such as optical nonlinearity. Currently, some progress has been made in research on shell-layer regulation of high-intensity optical properties in colloidal core/shell nanoplatelets [26,27,28]. In contrast, research on shell-layer regulation of high-intensity optical properties based on spherical quantum wells, especially the impact of the internal CdSe hole confinement layer on the material’s biexciton characteristics, optical nonlinearity, and other high-intensity optical properties, still requires in-depth exploration.

This study focuses on the influence of CdSe layer thickness on the high-intensity optical properties of CdS/CdSe/CdS SQWs. We prepared samples with varying CdSe layer thicknesses and investigated the effect of shell layer changes on biexciton binding energy using transient PL spectroscopy. The results showed that the biexciton binding energy varies within the range of −127 meV to −54 meV by adjusting the CdSe layer thickness. Furthermore, we conducted optical amplification studies on the samples using both one-photon and two-photon excitation modes, observing amplified spontaneous emission phenomena for both single excitons and biexcitons at thresholds as low as 12.4 μJ/cm^2^. Finally, we combined nonlinear optical measurements to analyze in depth the influence of shell structure on the optical amplification process.

## 2. Materials and Methods

### 2.1. Materials

The following chemicals were used as received without further purification or modification: Cadmium oxide (CdO, ≥99.99%), oleic acid (OA, 90%), sulfur powder (S, 99.999%), selenium powder (Se, 99.5%), and trioctylphosphine (TOP, 97%) were purchased from Sigma-Aldrich (St. Louis, MO, USA). 1-octadecene (ODE, 90%) and oleylamine (OLAM, 70%) were purchased from Alfa Aesar (Haverhill, MA, USA). 

### 2.2. Synthesis of Precursor

Synthesis of Cd Precursor (0.5 M)

First, 2.56 g (20 mmol) of CdO, 17.74 g of OA, and 15.78 g of ODE were loaded into a three-neck flask. The flask was heated to 110 °C and degassed for 30 min to remove residual water and oxygen. The temperature was raised to 250 °C and maintained for 1 h until a clear and nearly colorless solution was obtained. The reaction mixture was then cooled to 60 °C and transferred to a 40 mL glass vial.

Synthesis of S Precursor (0.25 M)

The S precursor was synthesized by combining 0.04 g of sulfur powder and 3.945 g of ODE in a 40 mL glass vial. The mixture was heated until the sulfur powder was completely dissolved, resulting in a homogeneous pale-yellow solution.

Synthesis of Se Precursor (0.5 M)

Se powder (0.48 g, 5 mmol) and TOP (8.3 g, 10 mL) were added to a 20 mL amber glass vial. The mixture was sonicated under light-protected conditions until Se fully dissolved.

### 2.3. Synthesis of CdS/CdSe/CdS SQWs

Synthesis of CdS Nanocrystals

The synthesis of CdS was adapted from the literature with some modifications [29]. First, 0.6 mL of Cd precursor (0.5 M) and 9 mL of ODE were degassed in a three-neck flask. The mixture was heated to 270 °C, followed by swift injection of 0.5 mL S precursor (0.25 M). The reaction proceeded at 250 °C for 8 min before quenching. The product was purified twice by ethanol and centrifugation, then redispersed in hexane.

Synthesis of CdS-CdSe Core-Shell NCs

The formation of the CdSe-emitting shell layer onto CdS-core NCs was performed by injection of two precursors (0.1 M Cd-oleate and 0.1 M TOP-Se, not mixed) via a syringe pump [30]. To begin, 80 nmols of CdS NCs were loaded into a 50 mL flask with 4 mL of OLAM and 10 mL of ODE and placed under argon, and the temperature was set to 240 °C. The desired amount of mixed Cd and Se precursor was added into the reaction flask at a rate of 1 mL/h. After injection, the reaction continued for 1 h at 240 °C. Then, it was removed from the heating mantle and left to cool to room temperature. The solution was cleaned by precipitation with 1:2 ethanol acetone mixture. The final NCs were suspended in chloroform.

Synthesis of CdS-CdSe-CdS Core-Shell-Shell SQWs

The growth of the CdS shell layer was adapted from the literature with some modifications [31]. The 100 nmols of CdS/CdSe prepared previously were loaded into a 50 mL flask with 2 mL of OLAM and 5 mL of ODE. The flask was degassed at 110 °C and then placed under argon and heated to 240 °C. Next, 0.1 M Cd and S precursors were alternately injected over a period of 5 min, followed by a 20 min reaction. Each injection cycle constituted one layer, with the growth temperature increasing by 20 °C per layer up to 300 °C. After the final precursor injection and 20 min reaction, the temperature was lowered to 280 °C for a 1 h annealing process and allowed to cool to room temperature. SQWs were then cleaned with acetone/methanol via centrifugation. The final SQWs were dispersed in hexane.

### 2.4. Characterization

Transmission electron microscopy (TEM) was performed using a JEM-2100 (JEOL Ltd., Tokyo, Japan). X-ray diffraction was performed using a Rigaku Ultima IV with samples drop-cast on to a miscut silicon sample holder with copper k-alpha X-rays. Absorption and PL spectra of samples were measured with a UV-3600 UV–vis spectrometer (Shimadzu Corporation, Kyoto, Japan) and a RF-5301PC (Shimadzu Corporation, Kyoto, Japan). The PL relaxation curves and quantum yields were measured using an FluoroMax fluorescence spectrometer (HORIBA Scientific, Palaiseau, France) equipped with an integrating sphere. Excitation was provided by a xenon lamp light source.

### 2.5. Single-Dot Spectroscopy

The single-dot PL traces were measured with a confocal microscope (Nikon Corporation, Tokyo, Japan). The SQWs samples were diluted in hexane and spin-coated on a quartz cover glass. The excitation light source was an EXR-15 Picoseconds pulsed laser (490 nm, 4.9 MHz) from NKT Photonics (Birkerød, Denmark). The excitation light was focused on the sample through a 100× oil lens, and the PL signal was collected through the same oil lens and sent to a CCD camera for PL spectrum measurement after passing through a 0.5 m spectrometer. The single-point PL signal could also be sent to two APDs through a non-polarization-selective 50/50 beam splitter for time-dependent single-photon counting measurement. The minimum time resolution of time-dependent single-photon counting was about 250 ps.

### 2.6. Transient PL Measurements

Transient emission spectra and PL decay curves were measured using a C5680 streak camera (Hamamatsu Photonics, Shizuoka, Japan). The excitation source was a Coherent Legend-F-1k femtosecond laser (800 nm, 150 fs, 1 kHz), with its output passed through a barium borate (BBO) frequency-doubling crystal to generate 400 nm high-energy femtosecond laser pulses as the pump source. The PL signals were measured under various excitation power densities ranging from 0 to 16 W cm^−2^. The excitation power was adjusted using a continuously variable neutral-density filter.

### 2.7. ASE Measurements

ASE spectra were measured using strip-shaped pulse excitation (light source same as Transient PL Measurements) and a Spectrapro-300i optical multichannel analyzer (Acton Research, now part of Princeton Instruments, Trenton, NJ, USA) for collection. Undiluted SQWs solution was spin-coated onto quartz substrates at a relatively low speed of 1000 rpm to obtain a thicker film. The aforementioned Coherent 800 nm femtosecond laser and its frequency-doubled 400 nm femtosecond laser were transformed into strip-shaped pulse light using a cylindrical prism and then vertically incident on the surface of the SQWs, forming a simple planar waveguide structure. The energy density of the excitation light was adjusted using a continuously variable neutral-density filter. An Acton Research Spectrapro-300i optical multichannel analyzer was used to collect and measure the emission spectra at the edge of the waveguide.

### 2.8. Nonlinear Optical Measurements 

The third-order nonlinear optical responses of the SQWs samples were investigated using Z-scan technology. The laser source employed was a Coherent Legend-F-1k femtosecond laser (Coherent Inc., Santa Clara, CA, USA) with 800 nm, 150 fs, 1 kHz. After focusing through a lens, the beam waist radius of the light spot was 28.8 μm, with a peak power of 28.9 GW cm^2^. The sample concentration was controlled at ~1.6 × 10^−5^ mmol/mL. Both open-aperture and closed-aperture response curves were measured for different samples at the same power. The influence of the solvent and cuvette on the experimental results was eliminated. This setup allowed for the investigation of nonlinear optical properties while avoiding thermal effects due to the laser’s low repetition rate and short pulse duration.

## 3. Results and Discussions

**Figure 1:**
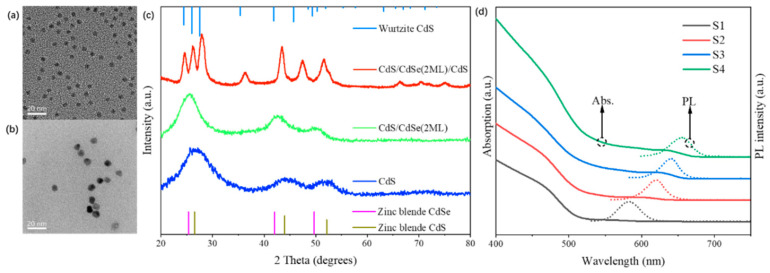
Structure and optical properties of SQWs samples. TEM images of (**a**) CdS/CdSe(2ML) core/shell NCs and (**b**) CdS/CdSe(2ML)/CdS SQWs. (**c**) XRD patterns of CdS core, CdS/CdSe(2ML) core/shell NCs, and CdS/CdSe(2ML)/CdS SQWs. (**d**) Absorption (solid lines) and photoluminescence (dashed lines) spectra of CdS/CdSe/CdS SQWs samples.

Figure 1 illustrates the structure and optical properties of the SQWs samples used in this study. We prepared CdS/CdSe/CdS SQWs with CdSe layer thicknesses ranging from one to four monolayers (ML). Figure 1a,b present TEM images of CdS/CdSe(2ML) core/shell quantum dots and CdS/CdSe(2ML)/CdS SQWs, respectively (additional TEM images and size analysis provided in Supplementary Figure S1). A significant increase in sample size is observed, directly demonstrating the coating of CdSe and CdS shell layers [32]. Figure 1c shows the corresponding XRD patterns (additional XRD patterns available in Supplementary Figure S2). The results indicate that the CdS core exhibits a zinc blende structure. After CdSe shell growth, the diffraction peaks are positioned between those of zinc blende CdS and CdSe but closer to CdSe due to the larger volume fraction of the outer shell. Upon further coating with the CdS outer shell, the sample’s diffraction peaks transition from zinc blende-characteristic peaks to typical wurtzite-characteristic peaks, possibly due to the higher stability of the wurtzite structure at high temperatures, leading to the growth of the outer wurtzite CdS shell [33,34]. Figure 1d presents the absorption and PL spectra (absorption and PL curves of intermediate samples shown in Supplementary Figure S3). Both absorption and PL spectra exhibit significant red shifts, arising from changes in the band structure due to shell coating and the attenuation of quantum confinement effects in the CdSe layer with increasing shell thickness, the latter resulting in a decrease in the CdSe layer bandgap. Furthermore, all samples display narrow-band emission characteristics in their PL spectra, indicating high size uniformity of the synthesized samples. 

The PL decay curves and quantum yields of samples S1–S4 under low-power excitation were measured using a fluorescence spectrometer. The decay curves, their fitting methods, and results are shown in Supplementary Figure S4. The fitting results and corresponding photoluminescence quantum yields (QY) of the samples are summarized in Table 1. Evidently, sample S1 exhibits three PL decay channels, while samples S2–S4 only show two distinct decay channels. Moreover, the weight of the fastest decay component τ_1_(%) demonstrates a high correlation with PLQY. Therefore, we conclude that the decay component (τ_1_) corresponds to the band-edge transition of single excitons, while the decay component (τ_2_) corresponds to defect-assisted emission processes [35,36]. Regarding the third decay component (τ_3_) in sample S1, considering its relatively low PLQY performance, we speculated that τ_3_ might be attributed to a new type of defect contribution different from the aforementioned defects [20,37]. As reported by Huang et al. [38] in their study on CdSe/CdS core/shell quantum dots, different defects can contribute differently to fluorescence decay. Through subsequent blinking tests, we further postulated that these could be internal defects in the CdSe shell layer.

**Figure 2:**
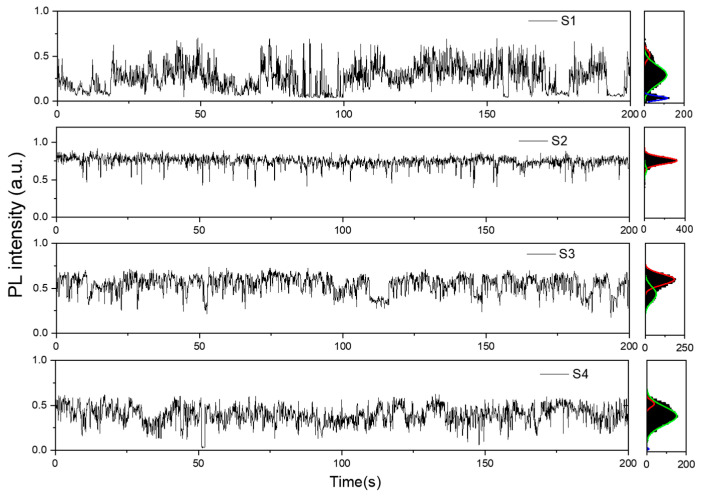
Single-dot PL intensity trajectories of SQWs. The measurements were performed with a bin time of 10 ms. The histogram on the right shows the statistical distribution of the intensity of the curve and the results of multi-peak fitting. The red line represents the “ON” state, the green line represents the “Int” state, and the blue line represents the “OFF” state.

To further investigate the relationship between defects, quantum yield, and photostability of SQWs, we conducted time-resolved PL blinking experiments on single SQWs for samples S1−S4 (Figure 2). Through tri-peak fitting analysis of the PL intensity distribution, we categorized the emission states of SQWs into “ON” state (normal emission), “OFF” state (non-emissive), and “Int” state (intermediate state). In sample S1, a significant “OFF” state (16.80%) was observed, which can be attributed to internal defects caused by non-uniform growth of the single-layer CdSe [39]. In contrast, S2 and S3 showed almost no “OFF” state, indicating that two or more layers of CdSe can effectively repair internal defects and suppress Auger recombination. However, when the CdSe shell thickness becomes excessive (as in sample S4), the proportion of the “Int” state increases, and a small amount of “OFF” state reappears (1.02%). Referencing the analysis by Allemand et al. [40], the “Int” state may correspond to charged exciton states in the sample under high-power excitation. The reappearance of the “OFF” state could be attributed to the effects introduced by interface defects. These findings demonstrate the impact of CdSe shell thickness on the optical properties of SQWs. When the shell is too thin, non-uniform growth easily introduces internal defects, while excessive thickness may lead to the formation of interfacial defects. Both scenarios are unfavorable for suppressing Auger recombination processes. 

**Figure 3:**
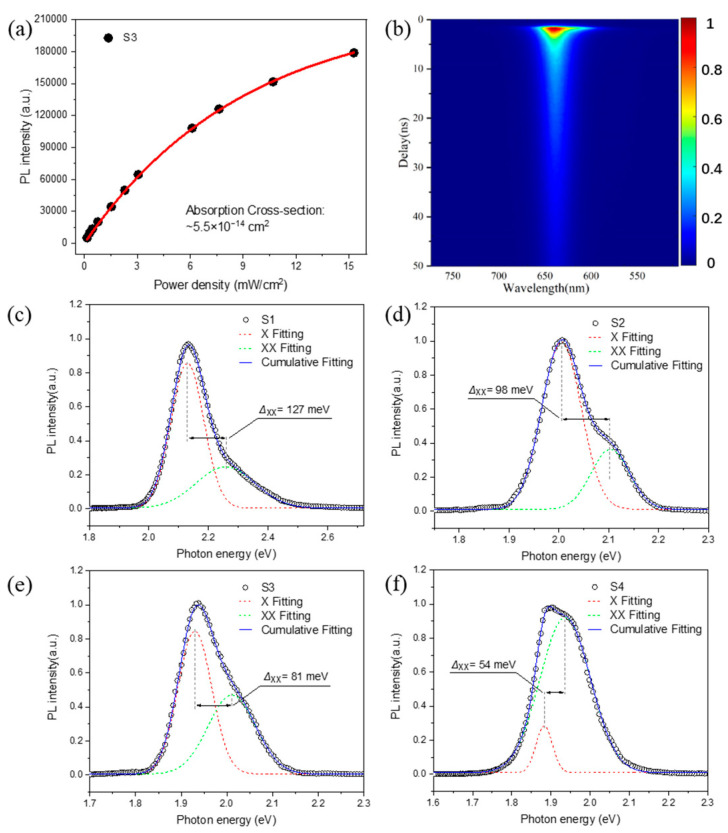
(**a**) PL intensity of sample S3 as a function of laser power density. Black dots represent experimental data, and the red line shows the fitting result. (**b**) Time-resolved PL spectrum of S3. Initial transient PL spectra of samples (**c**) S1, (**d**) S2, (**e**) S3, (**f**) S4 and their corresponding two-peak fitting results.

To investigate the multiexciton characteristics of SQWs under high-intensity optical excitation, we conducted transient PL spectroscopy measurements on the samples under 400 nm femtosecond pulse excitation. Using the PL saturation method [31] (Figure 3a, Supplementary Figure S5), we determined the one-photon absorption cross-sections for samples S1–S4 to be 6.8 × 10^−14^ cm^2^, 6.2 × 10^−14^ cm^2^, 5.5 × 10^−14^ cm^2^, and 5.0 × 10^−14^ cm^2^, respectively. Subsequently, the average exciton number $\langle N\rangle$ was calculated using the relation $\langle N\rangle =\sigma_{3.1eV}\times j$, where $\sigma_{3.1eV}$ is the one-photon absorption cross-section at 3.1 eV, and j is the excitation energy density. We then derived the required excitation power density while keeping the average exciton number $\langle N\rangle$ constant. Transient PL measured at $\langle N\rangle =$ 1.5 (Figure 3b, Supplementary Figure S6) showed significant PL peak broadening and bimodal characteristics, corresponding to single-exciton (X) and biexciton (XX) PL [41]. Through two-peak fitting analysis (Figure 3c–f), we calculated the exciton–exciton interaction energy ${∆}_{XX}$ for the four samples. The results indicate that all samples exhibit positive ${∆}_{XX}$ values, corresponding to exciton–exciton repulsion under type II or quasi-type II band alignment [42]. As the CdSe layer thickness increases, the ${∆}_{XX}$ value decreases, indicating an enlarged exciton distribution space and weakened mutual repulsion. Moreover, the positive ${∆}_{XX}$ implies a negative biexciton binding energy, suggesting that additional energy is required to form biexcitons, and thicker CdSe shells require less energy. This finding suggests that appropriately increasing the CdSe shell layer is beneficial for developing multiexciton applications of SQWs.

Based on the comprehensive results of time-resolved PL blinking experiments and transient PL measurements, samples S2 and S3 show great potential for efficient multiexciton applications. Therefore, we further investigated the PL decay characteristics of these two samples under high-power excitation (see Supplementary Figure S7). By fitting the time-dependent PL intensity curves with biexponential decay functions, we obtained the following results: For sample S2, the single-exciton PL lifetime $\tau_{X}$ is 24.5 ns, the biexciton PL lifetime $\tau_{XX}$ is 1.3 ns, and the corresponding biexciton quantum yield $QY_{XX}$ is 21.2%. Sample S3 exhibits even better performance, with $\tau_{X}$ of 25.2 ns, $\tau_{XX}$ of 1.6 ns, and a $QY_{XX}$ of 25.4%. Further calculations show that the biexciton Auger lifetimes $\tau_{Auger}$ for S2 and S3 are 2.52 ns and 4.54 ns, respectively (see Supplementary Materials for detailed calculations) [25]. This lifetime still falls short of the best reported results in the literature. This discrepancy may be attributed to the relatively small size of the CdS core and the absence of additional shell layers for surface passivation [25]. Evidently, the biexciton lifetime of SQWs has significantly improved compared to traditional quantum dots (which are typically on the picosecond scale). This enhancement is mainly attributed to the CdSe layer being distant from the core, resulting in increased quantum confinement volume and the exciton–exciton repulsion caused by negative biexciton binding energy, which effectively suppresses multiexciton Auger recombination and prolongs biexciton lifetime. Notably, sample S3 with a thicker CdSe layer demonstrates superior performance in suppressing Auger recombination and extending biexciton lifetime.

**Figure 4:**
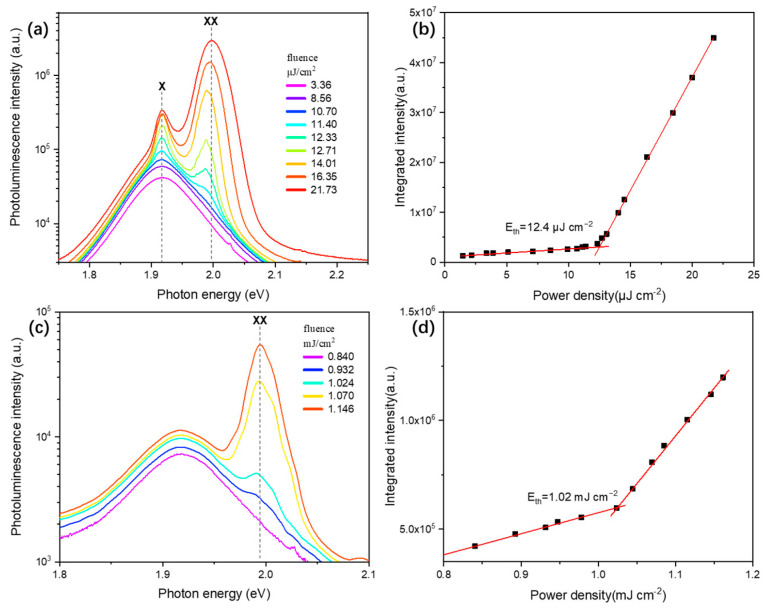
(**a**) Evolution of ASE spectra for typical sample films with increasing pump fluence, measured under stripe excitation at 400 nm. (**b**) Pump fluence dependence of the integrated intensity from (**a**). (**c**) Evolution of ASE spectra for typical sample films with increasing pump fluence, measured under stripe excitation at 800 nm. (**d**) Pump fluence dependence of the integrated intensity from (**c**).

Given the long Auger lifetime of SQWs, we conducted a detailed study of their ASE phenomena, including one-photon excitation at 400 nm and two-photon excitation at 800 nm. Consistent with previous experimental results, we observed the most significant ASE phenomenon in films prepared from sample S3. The ASE measurement results for the remaining samples are shown in Figures S8 and S9. Figure 4 shows the typical results of the evolution of ASE spectra under different pump intensities and the pump intensity dependence curve of PL intensity integral for sample S3. As shown in Figure 4a, under 400 nm excitation, two distinct ASE peaks were observed simultaneously as the excitation intensity increased, located at 1.918 eV and 1.997 eV, respectively. Based on previous analysis, we inferred that 1.918 eV corresponds to the single-exciton ASE peak, while 1.997 eV corresponds to the biexciton ASE peak, which is consistent with the previously measured biexciton binding energy of −81 meV. By analyzing the PL intensity integral at different pump intensities, we determined the ASE threshold under 400 nm one-photon excitation to be 12.4 μJ/cm^2^ (Figure 4b) with $\langle N\rangle =$1.36.

The ASE phenomenon of sample S3 under 800 nm two-photon excitation is shown in Figure 4c. Typically, the theoretical minimum threshold for single-exciton ASE should be lower than that for biexciton ASE [43]; however, our experimental observations do not align with this expectation. Under high-power excitation, the film sample primarily exhibited ASE deviating from the single-exciton peak, with its peak position essentially consistent with the biexciton ASE peak under one-photon excitation. This is the first observation of different ASE phenomena in SQWs under one/two-photon excitation. Similar phenomena were also observed in the one/two-photon-excited ASE of sample S2. Due to the lack of linear absorption in SQWs at 800 nm, exciton generation relies entirely on nonlinear absorption effects under high-power excitation, resulting in a significantly higher ASE threshold. As shown in Figure 4d, the ASE threshold under two-photon excitation (1.02 mJ/cm^2^) is nearly two orders of magnitude higher than that under one-photon excitation. This means that during two-photon pumping, in addition to two-photon absorption, part of the energy is also transferred to the film in the form of phonon vibrations through thermal effects. This helps spherical quantum wells overcome negative biexciton binding energy, which may be one of the reasons for preferentially observing biexciton ASE under two-photon excitation.

Referring to James et al.’s [21] analysis of the impact of film quality and reabsorption on the amplified spontaneous emission process and in combination with our experimental results, we speculated that high-power excitation at 800 nm may cause film damage, increasing losses and thus significantly raising the single-exciton ASE threshold. As the excitation power increases and reaches the biexciton ASE threshold, excitons in the SQWs are rapidly consumed by the biexciton ASE process, leading to our preferential observation of the biexciton ASE phenomenon. This is also consistent with our calculation that the average number of excitons corresponding to the ASE threshold under two-photon excitation ($\langle N\rangle =$1.58) is slightly higher than the result for one-photon excitation [44]. Our measurements and analysis of ASE in sample S4 (see Figure S9) also support this hypothesis. In stability tests of SQWs films, we observed that the small peak of single-exciton ASE disappeared after prolonged high-intensity testing, presumably due to the loss of sample and film layer quality under continuous excitation, further corroborating our hypothesis (Supplementary Figure S10).

**Figure 5:**
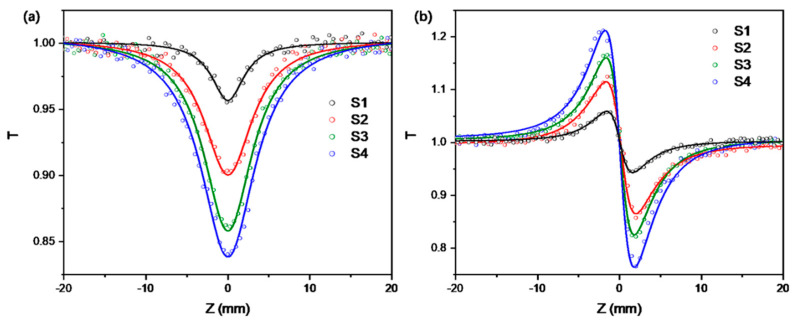
(**a**) Open-aperture Z-scan curves and (**b**) closed/open-aperture Z-scan curves of all samples. Points represent measured data, and lines show fitting results.

As exciton generation under two-photon excitation relies entirely on nonlinear absorption effects under high-power excitation, we further investigated the influence of CdSe thickness on the optical nonlinear characteristics of SQWs and analyzed its impact on the optical amplification process. We measured the third-order optical nonlinearity of samples S1–S4 under 800 nm femtosecond pulse excitation using Z-scan measurements. Figure 5a,b show the open-aperture Z-scan curves and closed/open-aperture Z-scan curves for all samples, respectively. From the open-aperture curves, we can observe that SQWs exhibit a valley shape under high-power excitation, representing the two-photon absorption phenomenon, with the nonlinear absorption effect increasing as the light intensity increases. In closed-aperture measurements, SQWs display a peak-then-valley phenomenon, suggesting a negative nonlinear refractive index under strong light, corresponding to a self-defocusing effect. Referring to our previous work on nonlinearity, we fitted the experimental results to obtain the two-photon absorption coefficient β and nonlinear refractive index coefficient n_2_ of the samples. For the normalized open-aperture curve, we used following Formula (1):

(1) $$\begin{array}{c} T\left(z\right)=1-0.33839\times \left[\frac{F_{TPA}}{1+x^{2}}\right]+0.13326\times {\left[\frac{F_{TPA}}{1+x^{2}}\right]}^{2}\\ -0.03446\times {\left[\frac{F_{TPA}}{1+x^{2}}\right]}^{3}+0.00377\times {\left[\frac{F_{TPA}}{1+x^{2}}\right]}^{4} \end{array}$$

where $F_{TPA}=\beta I_{0}L_{eff}$, and $L_{eff}=\left[1-\exp\left(-\alpha_{0}L\right)\right]/\alpha_{0}$. L is the actual thickness of the sample. $x=z/z_{0}$ represents the relative position of the sample, where $z_{0}=k\omega_{0}^{2}/2$ denotes the Rayleigh length of the beam, and $\omega_{0}$ is the beam waist radius.

For the normalized closed/open-aperture curve, we used following Formula (2):

(2) $$\begin{array}{c} T\left(z\right)=1+\frac{4\times \Delta \Phi_{0}\times x}{2^{0.5}\left(x^{2}+1\right)\left(x^{2}+9\right)}+\frac{4\times \Delta \Phi_{0}^{2}\times \left(3x^{2}-5\right)}{3^{0.5}(x^{2}+1{)}^{2}(x^{2}+9)(x^{2}+25)}\\ +\frac{32\times \Delta \Phi_{0}^{3}\times \left(x^{2}-11\right)x}{2(x^{2}+1{)}^{3}(x^{2}+9)(x^{2}+25)(x^{2}+49)} \end{array}$$

where $\Delta \Phi_{0}=k\gamma I_{0}L_{eff}$ represents the peak third-order nonlinear refractive phase shift on the axis.

Subsequently, the imaginary and real parts of the third-order nonlinear polarizability of the samples can be calculated using $Im\chi^{\left(3\right)}={cn_{0}}^{2}\lambda \beta /480\pi^{3}$ and $Re\chi^{\left(3\right)}=n_{0}n_{2}/3\pi$, respectively. Then, by applying $\chi^{\left(3\right)}=\sqrt{Re\chi^{{\left(3\right)}^{2}}+Im\chi^{{\left(3\right)}^{2}}}$ and $\sigma_{TPA}=hv\beta /N_{A}C$, we calculated their third-order nonlinear polarizability and two-photon absorption cross-section, respectively. The calculation results are shown in Table 2. The experimental results indicate that as the CdSe shell thickness increases, the real part of the third-order nonlinear polarizability $Re\chi^{\left(3\right)}$ decreases from −1.621 × 10^−13^ esu to −6.223 × 10^−13^ esu, reflecting an increase in the absolute value of the nonlinear refractive index, suggesting that the self-defocusing effect strengthens with increasing CdSe shell thickness. Simultaneously, the imaginary part of the third-order nonlinear polarizability $Im\chi^{\left(3\right)}$ increases from 0.278 × 10^−13^ esu to 1.258 × 10^−13^ esu, and the two-photon absorption cross-section $\sigma_{TPA}$ increases from 1.230 × 10^5^ GM to 5.567 × 10^5^ GM, indicating enhanced two-photon absorption capability of individual SQWs. However, the volume-normalized two-photon absorption cross-section shows a trend of first increasing and then decreasing, reaching a maximum value of 18.17 × 10^2^ GM/nm^3^ for sample S3, highlighting S3’s excellent performance in nonlinear absorption. This provides theoretical support for the S3 sample exhibiting the strongest amplified spontaneous emission phenomenon even under 800 nm two-photon excitation.

## 4. Conclusions

This study investigates the influence of CdSe layer thickness on the strong light properties of CdS/CdSe/CdS SQWs. By controlling the CdSe layer thickness (1–4 ML), we comparatively studied the evolution of various optical properties of SQWs with shell coating. The experiments showed that CdSe layers of 2 ML and above can effectively avoid internal defects caused by non-uniform growth, but excessively thick CdSe layers increase the generation of interfacial defects. As the CdSe layer thickness increases, the biexciton binding energy decreases from −127 meV to −54 meV, reflecting a weakening of exciton–exciton repulsion. The typical sample with a 3 ML CdSe shell exhibits relatively optimal biexciton characteristics, showing significant ASE phenomena under both one-photon (400 nm) and two-photon (800 nm) excitation, with thresholds of 12.4 μJ/cm^2^ and 1.02 mJ/cm^2^, respectively. Furthermore, different amplified spontaneous emission spectra were observed for the first time under one-photon and two-photon excitation. This phenomenon was explained from the perspectives of overcoming biexciton binding energy through thermal effects and changes in film losses. Subsequently, nonlinear optical measurements further revealed that the typical sample possesses a large volume-normalized two-photon absorption cross-section (18.17 × 10^2^ GM/nm^3^), favoring its two-photon excited optical amplification phenomenon. Therefore, by precisely controlling the CdSe layer thickness, we can effectively balance defect suppression, multiexciton characteristics, and nonlinear optical properties, laying the foundation for developing next-generation high-efficiency optoelectronic devices and nonlinear optical materials.

## Figures and Tables

**Table 1:** PL decay fitting results and QY of all final samples.

Sample	$A_{1}$	$\tau_{1}(ns)$	$A_{2}$	$\tau_{2}(ns)$	$A_{3}$	$\tau_{3}(ns)$	$\tau_{av}(ns)$	$\tau_{1}(\%)$	$\tau_{2}(\%)$	$\tau_{3}(\%)$	QY(%)
$S_{1}$	7.161	6.10	6.538	28.74	0.757	112.01	47.94	13.80	59.40	26.80	17.9
$S_{2}$	7.093	19.99	1.287	56.35	\	\	32.29	66.15	33.85	\	65.2
$S_{3}$	9.172	20.71	3.563	51.71	\	\	35.97	50.76	49.24	\	54.4
$S_{4}$	9.010	26.93	3.576	83.22	\	\	57.94	44.91	55.09	\	45.2

**Table 2:** Z-scan fitting results and related nonlinear parameters of all samples.

Sample	${\;n}_{2}/\;10^{-12}esu$	$\;\;\beta /\;10^{-12}m*W^{-1}$	$Re(\chi^{\left(3\right)})/\;10^{-13}esu$	$Im\left(\chi^{\left(3\right)}\right)/\;10^{-13}esu$	${\;\chi }^{\left(3\right)})/\;10^{-13}esu$	$\sigma_{2PA}/\;10^{5}GM$	$V_{total}/\;\;\;{nm}^{3}$	$\sigma_{2PA}/V_{total}/$ $10^{2}GM$ $*{nm}^{-3}$
$S_{1}$	−0.803	0.477	−1.621	0.278	1.644	1.230	138.5	8.878
$S_{2}$	−1.745	1.195	−3.520	0.696	3.588	3.081	192.2	16.03
$S_{3}$	−2.321	1.812	−4.681	1.056	4.799	4.673	257.2	18.17
$S_{4}$	−3.085	2.159	−6.223	1.258	6.349	5.567	335.4	16.60

## Data Availability

The original contributions presented in the study are included in the article and supplementary material; further inquiries can be directed to the corresponding authors.

## Supplementary Materials

The following supporting information can be downloaded at https://www.mdpi.com/article/10.3390/nano14191568/s1, Figure S1. TEM images of CdS/CdSe(xML)/CdS SQWs. (a–d) Samples S1–S4, x = 1–4 ML of CdSe, respectively. Scale bar: 20 nm; Figure S2. X-ray diffraction patterns of (a) CdS core, intermediate products coated with CdSe shells of different thickness, and (b) final products coated with about 4 ML CdS shells; Figure S3. Absorption and PL spectra of all transition samples. Solid lines represent absorption, and dashed lines represent PL; Figure S4. PL decay curves of (a) S1: CdS/CdSe (1ML)/CdS; (b) S2: CdS/CdSe (2ML)/CdS; (c) S3: CdS/CdSe (3ML)/CdS; and (d) S4: CdS/CdSe (4ML)/CdS quantum shells. Black dots represent experimental data, and red lines represent fitting results; Table S1. Relative Weights of ON, OFF, and INT States for Four Samples; Figure S5. PL intensity of (a) S1: CdS/CdSe (1ML)/CdS; (b) S2: CdS/CdSe (2ML)/CdS; (c) S3: CdS/CdSe (3ML)/CdS; and (d) S4: CdS/CdSe (4ML)/CdS SQWs measured with increasing laser power density. Black dots represent experimental data, and red lines represent fitting results; Figure S6. Time-resolved PL spectrogram of (a) S1: CdS/CdSe (1ML)/CdS, (b) S2: CdS/CdSe (2ML)/CdS, (c) S3: CdS/CdSe (3ML)/CdS; and (d) S4: CdS/CdSe (4ML)/CdS SQWs; Figure S7. PL decay curves and fitting of (a) S2 and (b) S3 SQWs; Figure S8 Evolution of ASE spectra for S2 films with increasing pump fluence, measured under stripe excitation at (a) 400 nm and (b) 800 nm; Figure S9 Evolution of ASE spectra for S4 films with increasing pump fluence, measured under stripe excitation at 400 nm; Figure S10. Stability of amplified spontaneous emission measured at excitation intensities of 40 μJ/cm^2^; Figure S11. TEM images of CdS/CdSe(2ML) NCs; Figure S12. The instrument response function (IRF) of the FluoroMax fluorescence spectrometer, where the inset shows a magnified portion from 0 to 100 ns.
