# Supplementary material for: Modulation of High-Intensity Optical Properties in CdS/CdSe/CdS Spherical Quantum Wells by CdSe Layer Thickness
Source: Nanomaterials (Basel). 2024 Sep 27;14(19):1568. doi: 10.3390/nano14191568 (PMC11478250; doi:10.3390/nano14191568)
Supplement: Supplementary file 1 [file nanomaterials-14-01568-s001.zip › nanomaterials-3210961-supplementary.pdf]

# Supporting Information for:

## Modulation of High-Intensity Optical Properties in CdS/CdSe/CdS Spherical Quantum Wells by CdSe Layer Thickness

Wenbin Xiang[1], Chunzheng Bai[1], Zhen Zhang[2], Bing Gu[1], Xiaoyong Wang[2], Jiayu zhang[1*]

1 Advanced Photonics Center, School of Electronic Science and Engineering, Southeast University, Nanjing, 210096, China; 230208149@seu.edu.cn (W.X); 230238398@seu.edu.cn (C,B); gubing@seu.edu.cn(B,G)

2 National Laboratory of Solid State Microstructures, College of Engineering and Applied Sciences, and School of Physics, Nanjing University, Nanjing 210093, China; 326645209@qq.com (Z.Z); wxiaoyong@nju.edu.cn (X.W)

* Correspondence: jyzhang@seu.edu.cn;

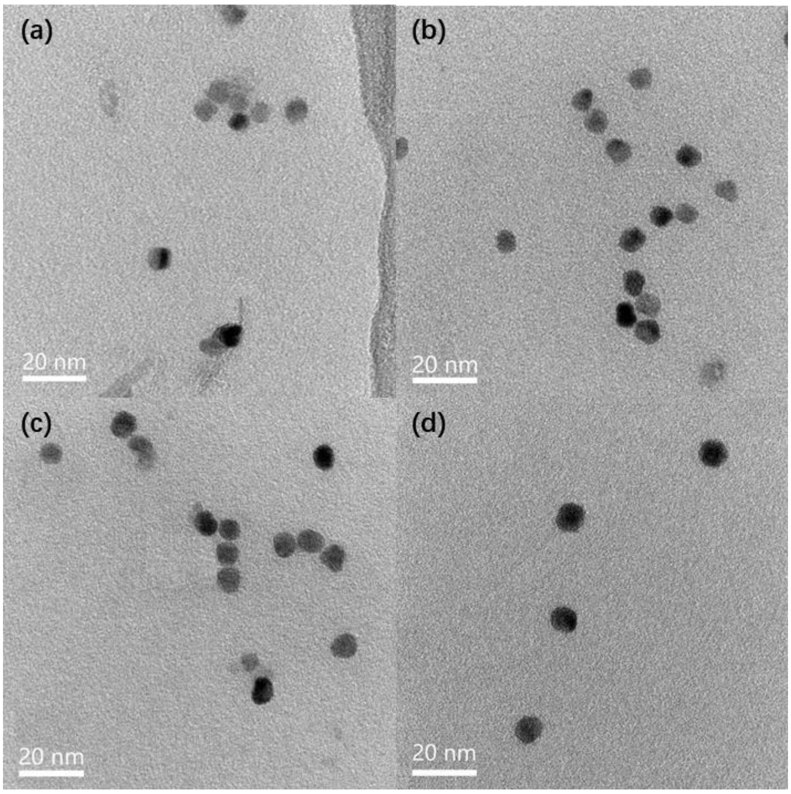


**Figure S1**. TEM images of CdS/CdSe(xML)/CdS SQWs. (a-d) Samples S1-S4, x = 1-4 ML of CdSe, respectively. Scale bar: 20 nm.

The core CdS nanocrystals were synthesized via a one-pot method. Due to their small size, conventional electron microscopy couldn't clearly resolve their dimensions. Following Peng et al.'s method[1], we estimated the CdS core diameter to be 2.96 nm using absorption spectroscopy and empirical equation (SE1).

$$D = (-6.6521\times 10^{-8})\lambda^3 + (1.9557\times 10^{-4})\lambda^2 - (9.2352\times 10^{-2})\lambda + 13.29 \quad (SE1)$$

Figure 1a shows a TEM image of CdS cores with 2 ML CdSe shell. The average diameter of CdS/CdSe(2ML) core/shell quantum dots was 4.26 ±0.43 nm, indicating a 0.65 nm increase per side, consistent with literature values for two CdSe monolayers[2]. CdS/CdSe core/shell quantum dots were further encapsulated with 4 ML CdS using successive ionic layer adsorption and reaction, yielding samples S1-S4. Figures S1 a-d show TEM images of S1-S4, with size distributions ranging from 6.42 ±0.55 nm (S1) to 8.43 ±0.76 nm (S4). Referring to the wurtzite CdS monolayer thickness (0.35 nm) reported by Xie et al.[3], the average diameter increase corresponds to approximately 4.14 CdS shell layers.

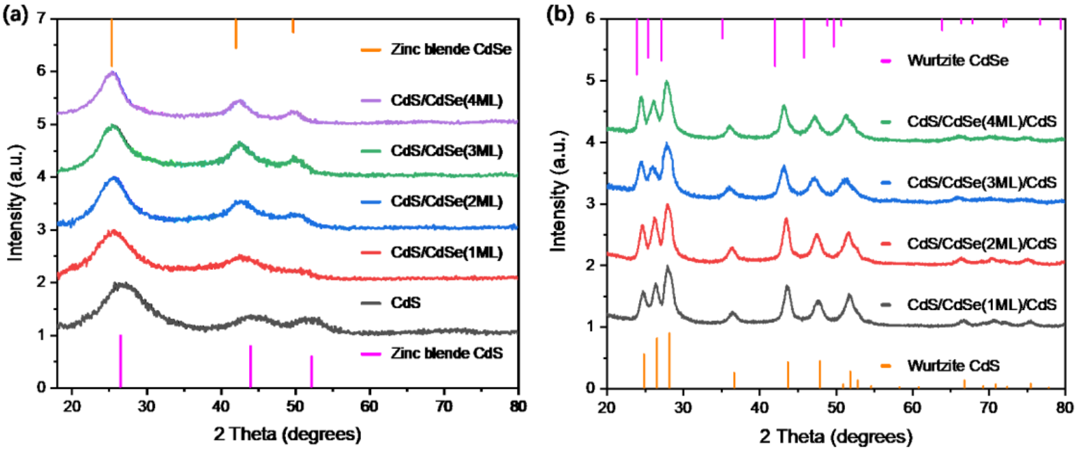


**Figure S2**. X-ray diffraction patterns of (a) CdS Core, intermediate products coated with CdSe shells of different thickness, and (b) final products coated with about 4 ML CdS shells.

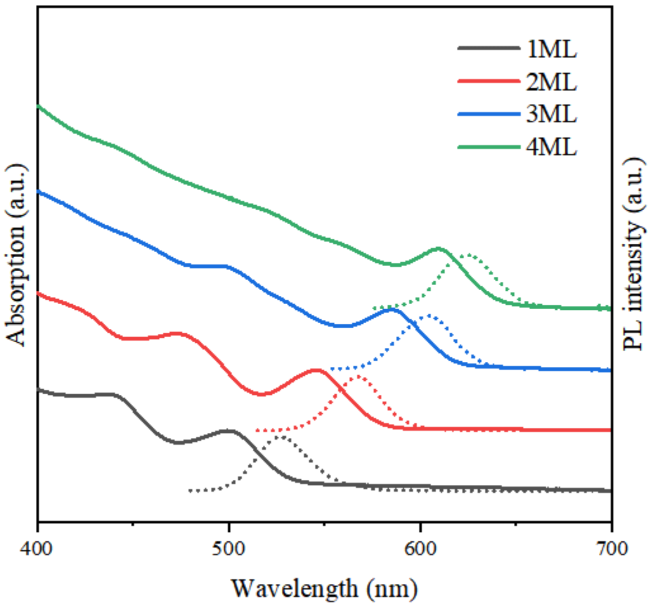


**Figure S3.** Absorption and PL spectra of all transition samples Solid lines represent to Absorption and dashed lines represent to PL.

Figure S3 displays absorption and PL spectra of CdS cores with 1-4 ML CdSe shells. All transition samples exhibit significant redshifts with increasing CdSe shell thickness. The first exciton absorption peak shifted from 386 nm (CdS core) to 609 nm, with diminishing redshifts for subsequent layers, consistent with electron-hole wavefunction expansion and reduced quantum confinement due to CdSe shell encapsulation.

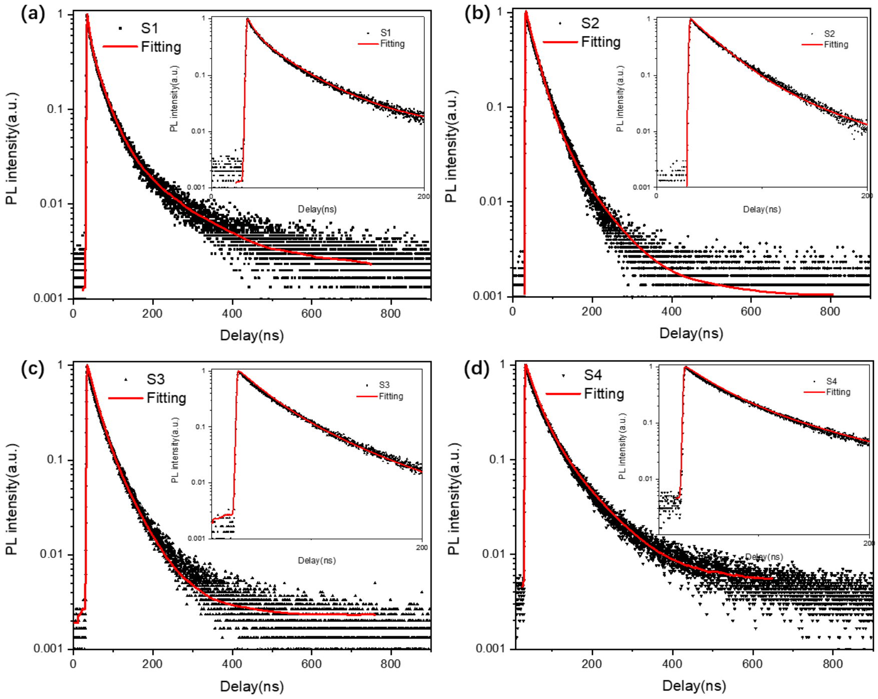


**Figure S4**. PL decay curves of (a) S1: CdS/CdSe (1ML)/CdS; (b) S2: CdS/CdSe (2ML)/CdS; (c) S3: CdS/CdSe (3ML)/CdS and (d) S4: CdS/CdSe (4ML)/CdS quantum shells. Black dots represent experimental data, and red lines represent fitting results. The insets provide a magnified view of the data from 0 to 200 ns.

For the data fitting in this section, we employed both triple-exponential and double-exponential decay models. Specifically, the fitting process was carried out using DAS6 software, which incorporates reconvolution with the instrument response function (IRF) based on the given fitting formulas. The general forms of the equations used are as follows:

For triple-exponential decay:

$$I(t) = A_1 * \exp\left(-t/\tau_1\right) + A_2 * \exp\left(-t/\tau_2\right) + A_3 * \exp\left(-t/\tau_3\right) + B$$

For double-exponential decay:

$$I(t) = A_1 * \exp\left(-t/\tau_1\right) + A_2 * \exp\left(-t/\tau_2\right) + B$$

Where $I(t)$ is the intensity at time t, $A_1$, $A_2$, and $A_3$ are amplitude coefficients, $\tau_1$, $\tau_2$, and $\tau_3$ are the respective decay time constants, and B is the background.

Below are the formulas for calculating the average lifetime for double-exponential and triple-exponential decay models:

Double-exponential decay model:

$$\tau_{av} = \frac{A_1\tau_1^2 + A_2\tau_2^2}{A_1\tau_1 + A_2\tau_2}$$

Triple-exponential decay model:

$$\tau_{av} = \frac{A_1\tau_1^2 + A_2\tau_2^2 + A_3\tau_3^2}{A_1\tau_1 + A_2\tau_2 + A_3\tau_3}$$

**Table S1**. Relative Weights of ON, OFF, and INT States for Four Samples

| Sample | ON | Int | OFF | ON/% | Int/% | OFF/% |
|---|---|---|---|---|---|---|
| S1 | 3.71 | 29.47 | 6.70 | 9.30 | 73.90 | 16.80 |
| S2 | 36.49 | 2.567 | \ | 93.43 | 6.57 | \ |
| S3 | 26.76 | 14.82 | \ | 64.36 | 35.64 | \ |
| S4 | 3.52 | 37.46 | 0.42 | 8.50 | 90.48 | 1.02 |

Table S1 is the relative weights of ON, OFF, and "Int" states for the four samples. Of these, the first three columns are the area parameters obtained from Gaussian fitting, while the last three columns represent their weights.

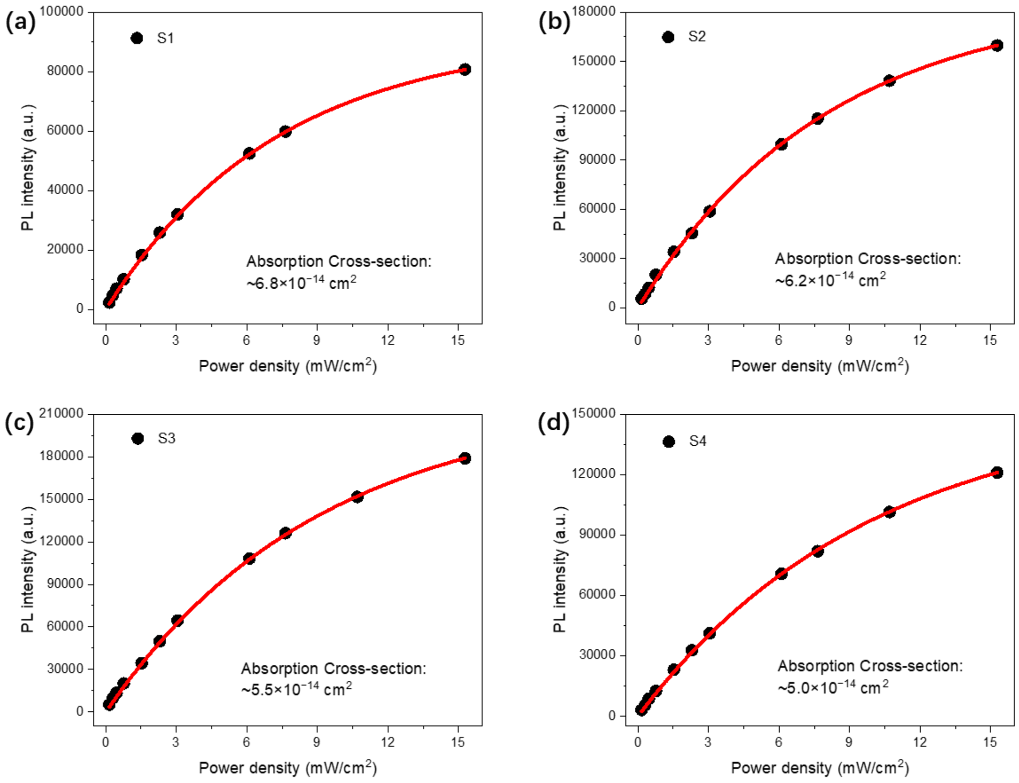


**Figure S5**. PL intensity of (a) S1: CdS/CdSe (1ML)/CdS; (b) S2: CdS/CdSe (2ML)/CdS; (c) S3: CdS/CdSe (3ML)/CdS; (d) S4: CdS/CdSe (4ML)/CdS SQWs measured with increasing laser power density. Black dots represent experimental data, and red lines represent fitting results.

For the data shown in Figure S5, the following equation can be used for fitting[4]:

$$I_{long} \propto 1 - e^{-j\sigma} \qquad (SE2)$$

Where j is the excitation energy density, $I_{long}$ is the PL intensity at a delay of 5 ns, and $\sigma$ is the single-photon absorption cross-section to be determined. By fitting with the above equation, we obtained single-photon absorption cross-sections for samples S1-S4 of $6.8\times10^{-14}$ cm$^2$, $6.2\times10^{-14}$ cm$^2$, $5.5\times10^{-14}$ cm$^2$, and $5.0\times10^{-14}$ cm$^2$, respectively. We observed that the single-photon absorption cross-

section does not increase with volume but instead shows a decreasing trend with increasing CdSe shell thickness. After fitting to obtain the single-photon absorption cross-section σ for the samples, we can substitute it into the following equation (SE3) to calculate the average exciton number $\langle \mathrm{N} \rangle$ under a certain excitation power:

$$\langle \mathrm{N} \rangle = \frac{\sigma P}{EF} \qquad (SE3)$$

Where P is the excitation power density, E is the excitation frequency, and F is the photon energy corresponding to the excitation wavelength. Since both E and F are constant for a given light source, when the absorption cross-section of the sample is determined, it can be assumed that the average exciton number is proportional to the excitation power density before saturation occurs.

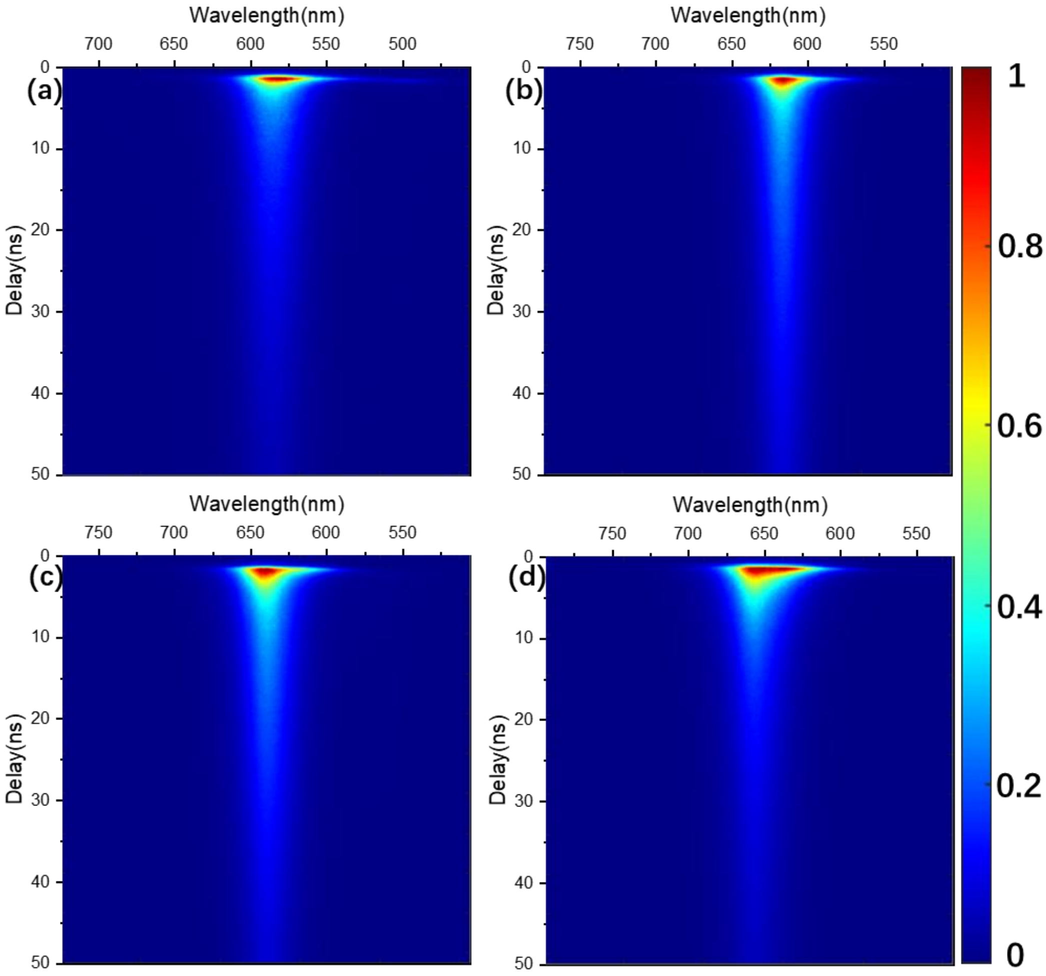


**Figure S6.** Time resolved PL spectrogram of (a) S1: CdS/CdSe (1ML)/CdS, (b) S2: CdS/CdSe (2ML)/CdS, (c) S3: CdS/CdSe (3ML)/CdS and (d) S4: CdS/CdSe (4ML)/CdS SQWs.

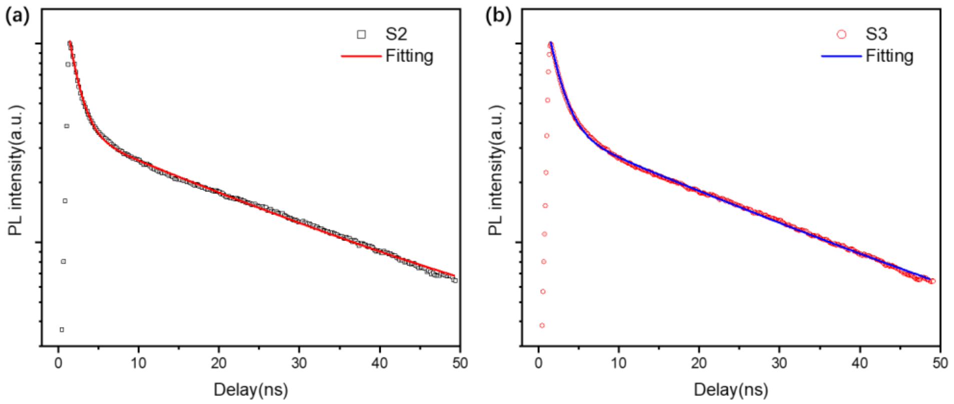


**Figure S7.** PL decay curves and fitting of (a) S2 and (b) S3 SQWs.

Figure S7 shows the intensity variation over time obtained by integrating transient PL data. These data were collected using a femtosecond laser and then integrated to obtain the PL intensity changes over time. The resulting curve was fitted using a biexponential decay model.

Using the single/biexciton lifetimes of the samples, we calculate their biexciton quantum yield $QY_{XX}$ according to the following equation [5]:

$$QY_{XX} = \frac{4\tau_{XX}}{\tau_X} \quad (SE4)$$

Subsequently, combining the single-exciton quantum yield $QY_X$ of the samples, we further calculate their biexciton Auger lifetime $\tau_{Auger}$. Here, we introduce a parameter $g_{XX} = QY_{XX}/QY_X$. The biexciton quantum yield then satisfies the following equation[6]:

$$QY_{XX} = \frac{\beta k_r}{\beta k_r + k_{Auger}} = g_{XX} QY_X \quad (SE5)$$

Where $k_r$ is the single-exciton radiative recombination rate, $k_{Auger}$ is the biexciton Auger recombination rate, and $\beta$ represents the factor by which the biexciton radiative rate increases compared to the single-exciton radiative rate. Further rearrangement yields:

$$\tau_{Auger} = \frac{1}{k_{Auger}} = \frac{g_{XX} QY_X}{\beta k_r (1 - g_{XX} QY_X)} = \frac{g_{XX}\tau_X}{\beta(1 - g_{XX} QY_X)} \quad (SE6)$$

To calculate the biexciton Auger lifetime $\tau_{Auger}$ of the sample at this point, we still need to obtain the value of $\beta$. For biexciton emission, if all de-excitation pathways involving two electron-hole pairs are open, then the radiative rate scales statistically with the number of electron-hole pairs m, yielding $\beta = 4$.

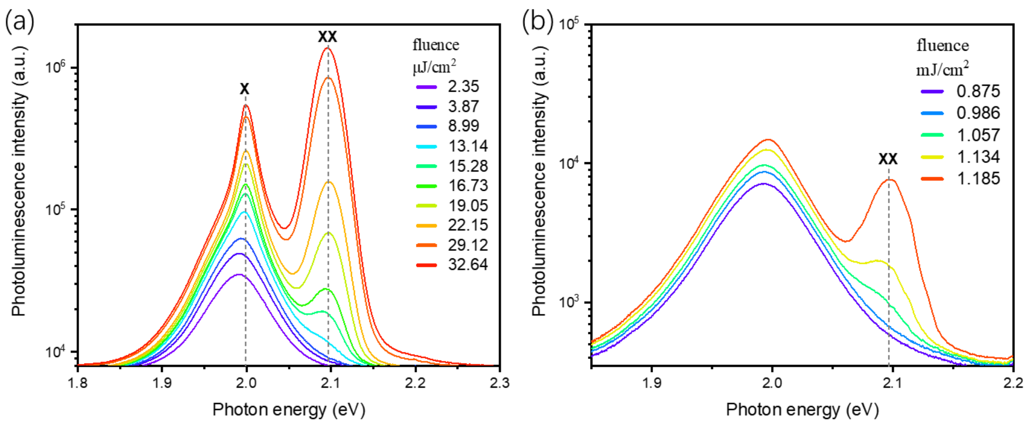


**Figure S8.** Evolution of ASE spectra for S2 films with increasing pump fluence, measured under stripe excitation at (a) 400 nm and (b) 800 nm.

Figure S8 shows the power-dependent evolution of single/two-photon excited ASE for sample S2. It should be noted that due to the extremely low PLQY of sample S1, no ASE phenomenon was observed, and thus no data could be provided. For sample S2, under single-photon excitation, we observed the simultaneous occurrence of single and biexciton ASE, similar to sample S3 described in the main text. However, the intensity ratio differs from that of S3, which may be related to the differences in $QY_x$ and $QY_{xx}$ between samples S2 and S3. Furthermore, under two-photon excitation, only biexciton ASE was observed, further supporting our analysis in the main text.

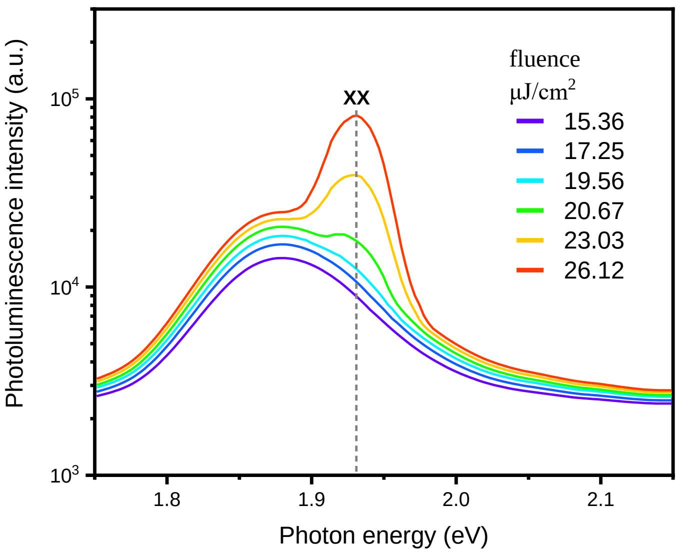


**Figure S9.** Evolution of ASE spectra for S4 films with increasing pump fluence, measured under stripe excitation at 400 nm.

Figure S9 shows the evolution of ASE under single-photon excitation for sample S4. It can be observed that the threshold required for ASE significantly increases, while the intensity weakens. This may result from increased defects enhancing the film layer loss, leading to a higher average number of excitons required for ASE. Additionally, the biexciton binding energy is further reduced, favoring biexciton ASE. Consequently, the single-photon excited ASE in sample S4 exhibits a

situation similar to the two-photon ASE of samples S2 and S3. As for two-photon excitation of sample S4, due to inherently greater losses and increased power-induced damage to the thin film, no significant ASE was observed.

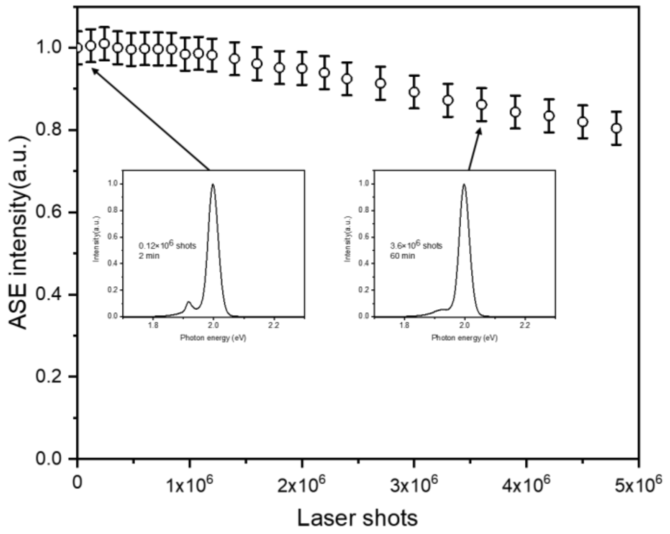


**Figure S10.** Stability of Amplified Spontaneous Emission measured at excitation intensities of 40 μJ/cm$^2$.

To verify the stability of the SQWs, we continuously pumped the SQWs film with a 400 nm femtosecond laser at an excitation power density of 40 μJ/cm$^2$. The PL spectra and intensity changes were continuously recorded using a Spectrapro-300i optical multichannel analyzer. The results are shown in Figure S10. The ASE from the SQWs film under high-power excitation is mainly contributed by biexciton emission. Its intensity remained above 80% of the initial intensity after continuous pumping for 1 hour. The inset shows the PL spectra after 2 minutes and 1 hour of testing. The small peak corresponding to single-exciton ASE disappeared after long-term high-intensity testing, which is presumed to be caused by the degradation of sample and film quality under continuous excitation, further corroborating our previous hypothesis.

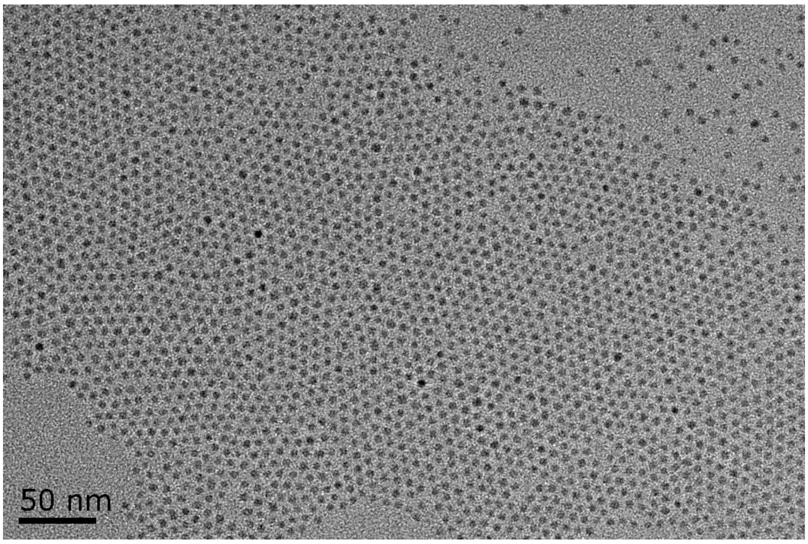

**Figure S11**. TEM images of CdS/CdSe(2ML) NCs.

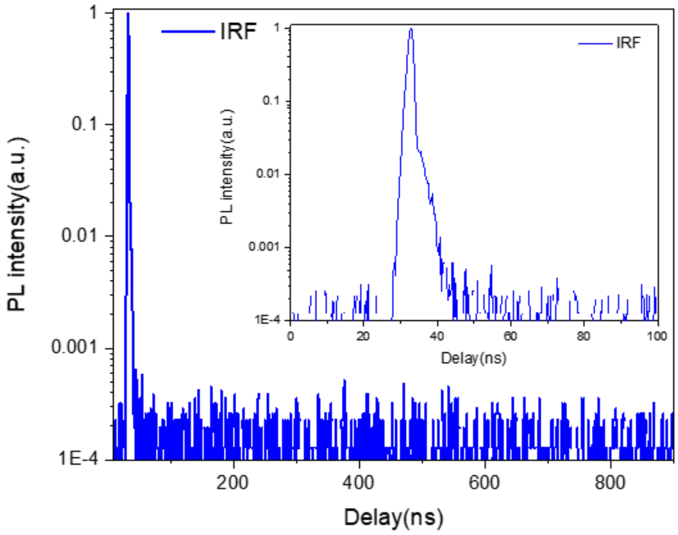


**Figure S12**. The instrument response function (IRF) of the FluoroMax fluorescence spectrometer, where the inset shows a magnified portion from 0 to 100 ns.

Reference


1. Yu WW, Qu L, Guo W, Peng X. Experimental Determination of the Extinction Coefficient of CdTe, CdSe, and CdS Nanocrystals. *Chemistry of Materials.* 2003/07/01 2003;15(14):2854-2860.
2. Jeong BG, Park Y-S, Chang JH, et al. Colloidal Spherical Quantum Wells with Near-Unity Photoluminescence Quantum Yield and Suppressed Blinking. *ACS Nano.* 2016/10/25 2016;10(10):9297-9305.
3. Xie R, Kolb U, Li J, Basché T, Mews A. Synthesis and Characterization of Highly Luminescent CdSe – Core CdS/Zn0.5Cd0.5S/ZnS Multishell Nanocrystals. *Journal of the American Chemical Society.* 2005/05/25 2005;127(20):7480-7488.
4. Zhang L, Li H, Liao C, et al. New Insights into the Multiexciton Dynamics in Phase-Pure Thick-Shell CdSe/CdS Quantum Dots. *The Journal of Physical Chemistry C.* 2018/11/01 2018;122(43):25059-25066.
5. Bae WK, Padilha LA, Park Y-S, et al. Controlled Alloying of the Core–Shell Interface in CdSe/CdS Quantum Dots for Suppression of Auger Recombination. *ACS Nano.* 2013/04/23 2013;7(4):3411-3419.
6. Harankahage D, Cassidy J, Beavon J, et al. Quantum Shell in a Shell: Engineering Colloidal Nanocrystals for a High-Intensity Excitation Regime. *Journal of the American Chemical Society.* 2023/06/21 2023;145(24):13326-13334.
